# Electronic polymers in lipid membranes

**DOI:** 10.1038/srep11242

**Published:** 2015-06-10

**Authors:** Patrik K. Johansson, David Jullesson, Anders Elfwing, Sara I. Liin, Chiara Musumeci, Erica Zeglio, Fredrik Elinder, Niclas Solin, Olle Inganäs

**Affiliations:** 1 Biomolecular and Organic Electronics, Department of Physics Chemistry and Biology, Linköping University, SE-58183, Linköping, Sweden; 2 Current address: National ESCA Surface Analysis Center for Biomedical Problems, Department of Bioengineering, University of Washington, Seattle, WA, US-98195, United States; 3 Current address: Systems and Synthetic Biology, Department of Chemical and Biological Engineering, Chalmers University of Technology, SE-41296, Gothenburg, Sweden; 4 Department of Clinical and Experimental Medicine, Linköping University, SE-58185, Linköping, Sweden

## Abstract

Electrical interfaces between biological cells and man-made electrical devices exist in many forms, but it remains a challenge to bridge the different mechanical and chemical environments of electronic conductors (metals, semiconductors) and biosystems. Here we demonstrate soft electrical interfaces, by integrating the metallic polymer PEDOT-S into lipid membranes. By preparing complexes between alkyl-ammonium salts and PEDOT-S we were able to integrate PEDOT-S into both liposomes and in lipid bilayers on solid surfaces. This is a step towards efficient electronic conduction within lipid membranes. We also demonstrate that the PEDOT-S@alkyl-ammonium:lipid hybrid structures created in this work affect ion channels in the membrane of *Xenopus* oocytes, which shows the possibility to access and control cell membrane structures with conductive polyelectrolytes.

Lipids have an extraordinary ability to form biomembranes and form therefore the compartments in living systems. This property of lipids can also be used *in vitro* to create a wide variety of model membrane systems (MMS) such as liposomes, nanotubes, monolayers, bilayers and multilayers. MMS are widely used in materials science to study catalysis, electrochemistry and protein interactions^1^. Electronic polymers may exist in semiconducting and luminescent forms, and also in metallic forms after doping—and the use of electronic polymers for photonic probing of biomembrane models is well established^2^, but not the electrical probes. The development of such probes to access electronic and ionic processes within biomembranes in a non-destructive manner may enable new modes to observe and control biological processes. The classical electrode methods^3^, from metal wires^4^,^5^ to patch clamp contacts^6^, can seriously disturb the biomembrane geometry and stability^7^.

Electric currents in living systems are with few exceptions^8^,^9^,^10^ transmitted via ionic currents. In contrast, currents in man-made high precision devices are typically carried by electrons. An interface between these requires electroactive materials, where ions and electrons can be released during signaling. It could be desirable to introduce this electronic contact and pathway within a biomembrane, thus utilizing the inherent electrical insulation already present due to the nonpolar biomembrane interior. A novel and intriguing approach to achieve such soft electrical interfaces would be to introduce electrically conducting materials into a lipid membrane. In such a scenario, the dimensions of the conductor should be found in the range of the thickness of biomembranes, limiting the choice of conductors to the nanoregime. Formation of nanowires from metals in the melt are subject to geometric instability as the Rayleigh-Plateau destabilization of a column into droplets may occur; similar phenomena have been reported also for nanowire growth by electrochemical reduction^11^. Carbon based conductors offer another possibility to form nanowires as well as nanosheets. Graphene and carbon nanotubes (CNTs) have the requisite thickness, and in particular CNTs have the requisite length for wiring within the biomembrane. However, the stiffness of CNTs poorly matches the flexibility of the biomembranes and they are known to perturb lipid membranes by penetration^12^. Also fullerenes are known to destabilize lipid membranes^13^.

In contrast, organic electronic polymers offer a better match of chemical and mechanical properties, and have the potential to be biocompatible^14^,^15^,^16^. The behavior of polypyrrole inserted into lipid membranes have been investigated electrochemically^17^. Previously, simulations showed that conducting molecular wires from poly(alkylthiophene) could penetrate through supported lipid membranes^18^, which suggested the possibility to use thiophene-based polymers for transporting currents across biomembranes. In more recent work, efforts to integrate semiconducting polyelectrolytes into biomembranes and cells have been reported^19^,^20^,^21^,^22^,^23^,^24^,^25^. The results demonstrate that the conductive polyelectrolytes can spontaneously integrate in cell membranes and assist in conduction across the biomembranes. However, no long-range conductivities within lipid bilayers have been reported yet.

Poly(3,4-ethylenedioxythiophene)sulfonate (PEDOT-S) is a thiophene-based polymer (Fig. 1a) that can exhibit metallic conductivities at 30 S/cm^26^,^27^. In PEDOT-S, polarons and bipolarons are the charge carriers responsible for the conductivity^26^,^28^,^29^,^30^. These are positive holes that form during doping when protons in the surrounding solution bind with the conjugated backbone. This makes it possible to tune the degree of doping with pH. One of the virtues with PEDOT-S is that it has negatively charged sulfonate groups with low pKa, covalently attached to the backbone, which stabilizes the polarons and bipolarons in the doped state of the polyelectrolyte and makes it by definition self-doped. We have previously used PEDOT-S to prepare conducting composite materials with DNA^31^ and proteins^32^. Its incorporation into structures based on phospholipids would provide a pathway for also creating conductive lipid nanotubes and membranes.

Conducting polymers/oligomers should be possible to integrate into lipid membranes. It has been shown that a fluorescent polyelectrolyte resembling PEDOT-S interacts with liposomes prepared from 1,2-dioleoyl-sn-glycero-3-phosphocholine (DOPC, Fig. 1a)^33^ that is one of the most common phospholipids in animal and plant cells. The backbone of PEDOT-S is hydrophobic and would thus naturally associate with the hydrophobic part of the lipid membrane; however, PEDOT-S is substituted by hydrophilic sulfonate groups making integration into the membrane challenging. At the same time these hydrophilic sulfonate groups make the polymer self-doped which may give the material unique characteristics if included into a biomembrane. Accordingly we sought ways of preparing supramolecular complexes between cations and the anionic PEDOT-S. Such complexes may have lipophilic properties, while they at the same time retain the possibility of self-doping. Polymer-surfactant mixtures have a rich supramolecular chemistry^34^ and previous reports have shown that alkyl-ammonium salts can be used to increase the hydrophobicity of DNA^35^,^36^, cellulose nanocrystals^37^ and other polyelectrolytes^38^,^39^,^40^,^41^. Inspired by this and one study on how to increase the surface activity of PEDOT:polystyrene-sulfonate^42^, we investigated various alkyl-ammonium salts to make hydrophobic PEDOT-S via supramolecular interactions. The formed PEDOT-S@alkyl-ammonium complexes could be dissolved in organic solvents. We characterized these complexes spectroscopically and electrically, and used the PEDOT-S@dioctylammonium complex together with DOPC to create PEDOT-S@dioctylammonium:DOPC lipid hybrid structures (denoted P-S@dioct:DOPC throughout this work). We investigated the structures by studying their adsorption behavior on lipid membranes with quartz crystal microbalance dissipation (QCM-D), and verified their electrical properties by measuring their conductivities on nanoelectrodes, as well as by conductive atomic force microscopy (C-AFM). We also demonstrated that the P-S@dioct:DOPC structures communicate with ion channels in biological cells by making them open at more negative membrane potentials.

## Results

### Preparation and Characterization of PEDOT-S@alkyl- ammonium Complexes

**Figure 1:**
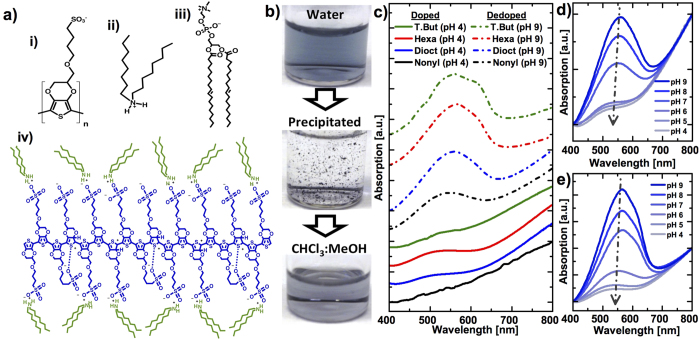
Characterization of the PEDOT-S@alkyl-ammonium complexes. (**a**) i) The monomer of PEDOT-S, ii) dioctyl-ammonium chloride, the alkyl-ammonium molecule used in subsequent experiments, iii) DOPC, the molecule used to create P-S@dioct:DOPC structures, iv) the presumed structure of the PEDOT-S@dioctyl-ammonium complex. (**b**) PEDOT-S is soluble in water (top), but precipitates in 2.5 mM dioctyl-ammonium (middle) and the precipitate is soluble in chloroform (bottom). (**c**) UV-vis spectra of PEDOT-S@alkyl-ammonium complexes dissolved (100 *μ*g/mL based on PEDOT-S), in chloroform:methanol (2:1) after being precipitated from water solutions at pH 4 (solid) or pH 9 (dashed), corresponding to doped and dedoped PEDOT-S respectively. The alkyl-ammonium salts used were tetrabutyl-ammonium (green), hexadecyl-trimethyl-ammonium (red), dioctyl-ammonium (blue) and nonyl-ammonium (black). PEDOT-S@dioctyl-ammonium (**d**) and PEDOT-S@hexadecyl-trimethyl-ammonium (**e**) complexes dissolved (100 *μ*g/mL based on PEDOT-S) in chloroform:methanol (2:1) were characterized after being precipitated from water solutions with pH in the range 9–4. The arrows indicate increased doping as the pH is decreased.

PEDOT-S interacts poorly with DOPC membranes, but interaction can be promoted by forming a supramolecular complex between the polyelectrolyte and amphiphilic cations that interact with the anionic sulfonate groups. We thus investigated the interaction between PEDOT-S (~16 monomer units^43^) and various alkyl-ammonium salts, with the aim to form PEDOT-S@alkyl-ammonium complexes that are soluble in organic solvents (Fig. 1b). This would provide an attractive route to incorporate PEDOT-S in liposomal membranes by mixing the hydrophobic PEDOT-S@alkyl-ammonium complexes with the phospholipids directly in the solvent used when forming the liposomes. Many of the investigated alkyl-ammonium salts formed complexes with PEDOT-S, which precipitated in water; however, not all of the precipitates were readily dissolved in the organic solvent. UV-vis spectroscopy can be used to verify that PEDOT-S remains in its doped state after forming the complex, since it has distinct spectral appearances in its doped and dedoped state^44^, where dedoping is indicated by a broad absorption peak at about 500–600 nm. Four of the alkyl-ammonium salts tested in this work (nonyl-ammonium chloride, tetrabutyl-ammonium fluoride, hexadecyl-trimethyl-ammonium bromide and dioctyl-ammonium chloride) created complexes with PEDOT-S having various degrees of solubilities in chloroform:methanol (2:1). We found that the doping of PEDOT-S in the complexes was preserved in the organic solvent, which made it possible to control the degree of doping with the pH of the water solution from which the PEDOT-S@alkyl-ammonium complexes were precipitated (Fig. 1c). Dioctyl-ammonium chloride and hexadecyl-trimethyl-ammonium bromide were the easiest to work with and gave precipitates readily dissolvable in organic solvents. For both molecules, it was possible to tune the degree of doping by adjusting the pH of the water solution (Fig. 1d,e). Pure PEDOT-S exhibits conductivities at about 30 S/cm and to verify that the mixing of PEDOT-S with insulating alkyl-ammonium molecules does not completely diminish this conductivity, we blade coated thin films of the complexes on microscope glass slides. I-V curves of the films revealed that the complexes prepared at pH 4 still exhibited conductivities at about 0.4 S/cm and 0.8 S/cm for fully doped PEDOT-S@dioctylammonium and PEDOT-S@hexadecyl-trimethyl-ammonium films, respectively. In all the subsequent experiments presented below, we chose to use the PEDOT-S@dioctyl-ammonium complex.

### Interactions of P-S@dioct:DOPC Structures with Lipid Bilayers

**Figure 2:**
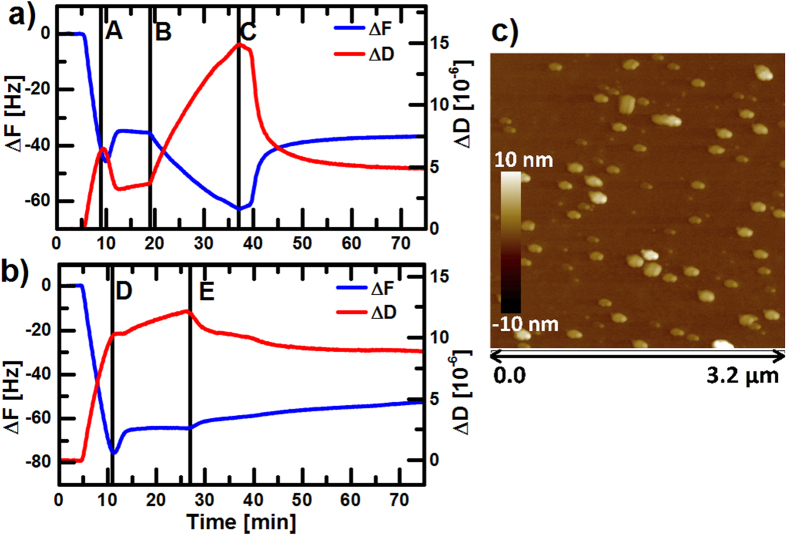
Characterization of P-S@dioct:DOPC interactions with supported lipid bilayers. (**a**) 100 nm DOPC liposomes (500 *μ*g/mL) reach the SiO_2_ QCM-D sensor at 6 min and a lipid bilayer is formed at about 9 min (**A**). P-S@dioct:DOPC structures (500 *μ*g/mL DOPC, 50 *μ*g/mL PEDOT-S) were introduced and reached the sensor at 19 min (**B**) which caused shifts in frequency and dissipation indicative of structure adsorption. After additional 18 min, rinsing with pure PBS started (**C**) and the curves returned to the values for a clean lipid bilayer. **b**) P-S@dioct:DOPC structures (500 *μ*g/mL DOPC, 50 *μ*g/mL PEDOT-S) were introduced directly and reached the SiO_2_ QCM-D sensor at 5 min. A lipid bilayer was formed at about 11 min (**D**) and the shifts in dissipation and frequency remained high, which indicated presence of P-S@dioct:DOPC structures on the surface. Rinsing with PBS started after additional 16 min (**E**) and continued for 50 min, but the shifts of the curves remained high. **c**) AFM image of the P-S@dioct:DOPC structures adsorbed onto a supported lipid bilayer prepared on a SiO_2_ substrate.

There are many protocols for preparing liposomes and we used one involving extrusion, which results in small unilamellar vesicles with controllable diameters. By including the hydrophobic PEDOT-S@dioctyl-ammonium complex when preparing the liposomes, it was possible to create structures based on DOPC with PEDOT-S included. To further characterize these structures, we performed quartz crystal microbalance with dissipation (QCM-D) studies on their interaction with lipid bilayers. First, DOPC liposomes were transported through the microfluidic system to the SiO_2_ sensor surface and a lipid bilayer was formed^45^,^46^,^47^. When the solution with the P-S@dioct:DOPC structures were introduced, shifts in frequency and dissipation were observed, indicating adsorption of the structures onto the lipid bilayer (Fig. 2a). Subsequent rinsing with phosphate buffered saline (PBS) made the QCM-D graphs return to the values for the pure lipid bilayer, which shows that the adsorbed structures were easily removed. Second, we introduced a liposome solution with P-S@dioct:DOPC structures directly to the SiO_2_ sensor surface, without first creating a lipid bilayer. This time, the QCM-D graphs indicated that a lipid bilayer was formed, but the frequency and dissipation stayed at rather high values, which indicates that larger structures are present in or on the bilayer already from the beginning (Fig. 2b). The frequency and dissipation shifts remained at rather high values after rinsing with PBS. This shows that there are P-S@dioct:DOPC structures embedded in the lipid bilayer. AFM measurements were made on samples prepared as in the first QCM-D experiment, but with only 30 s rinsing with MilliQ water to remove salts. This showed adsorbed structures on the bilayer, with a diameter of about 100 nm (Fig. 2c), which agrees well with the 100 nm orifices used in the liposome extrusion process. The heights were about 10 nm, which probably is due to collapse of the structures during drying. It is thus demonstrated that lipids with associated complexes including PEDOT-S can attach and possibly integrate into bilayers on a solid surface.

### Electrical Characterization of P-S@dioct:DOPC Structures

**Figure 3:**
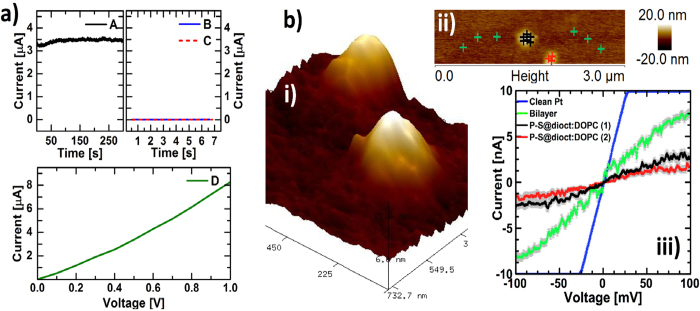
Electrical characterization of P-S@dioct:DOPC structures. **a**) Supported lipid bilayers were prepared on nano-electrodes with 100 nm gaps, and 100 nm P-S@dioct:DOPC structures (500 *μ*g/mL DOPC, 50 *μ*g/mL PEDOT-S) prepared at pH 7.4 were then adsorbed. A time-series during 300 s with 0.5 V applied voltage show stable electronic currents (**A**). References with either only lipid bilayers (**B**) or lipid bilayers treated with PEDOT-S dissolved in MilliQ (100 *μ*g/mL) during 15 min (**C**) were not conductive and the measurements were terminated after 7 s. (**D**) shows a voltage sweep for the P-S@dioct:DOPC structures between 0–1 V. **b)** 400 nm P-S@dioct:DOPC structures (500 *μ*g/mL DOPC, 50 *μ*g/mL PEDOT-S) were applied on Pt substrates and measured on by C-AFM. i) shows two representative 400 nm structures that had collapsed on the surface and had heights of 17 nm and 21 nm respectively. ii) shows the various spots that were measured and iii) shows the average currents in voltage sweeps between −100 to 100 mV for the two structures (17 nm black, 21 nm red), the surrounding lipid bilayer (green) and the clean Pt substrate (blue).

We measured currents through P-S@dioct:DOPC structures adsorbed on lipid bilayers prepared on nanoelectrodes with 100 nm gaps that matched the size of the structures. Voltage sweeps between 0–1 V and a 300 s time-series at 0.5 V showed currents in the range of a few *μ*A (Fig. 3a). The stable current in the time series shows that the conductance is electronic. Reference samples with only lipid bilayers, or lipid bilayers treated with hydrophilic PEDOT-S dissolved in MilliQ water with subsequent rinsing during 30 s with pure MilliQ water, did not exhibit any conductivities. To demonstrate that the P-S@dioct:DOPC can be responsible for the measured currents, we also performed C-AFM. For this, larger P-S@dioct:DOPC structures were prepared by using 400 nm, orifices in the extrusion process, as it otherwise would have been difficult to achieve the resolutions required for reliable results. We used a Pt metal film as conductive substrate, on which the P-S@dioct:DOPC structures were adsorbed, as in the second QCM-D experiment. The C-AFM revealed 400 nm structures that had collapsed and were about 20 nm in height (Fig. 3b). With applied voltages of up to 100 mV, it was possible to measure a few nA through these structures (Fig. 3b), which would not have been possible if they were not conductive, as the heights exceed the quantum tunneling range. The low thickness of the surrounding lipid bilayer, however, allows tunneling, which can explain the higher currents for these spots. A rough estimation, based on the electrical measurements and sample geometries with the nanoelectrodes (5 nm bilayers on Au-finger electrodes having 10 fingers á 20 *μ*m from each side with 100 nm gaps) yield average lateral conductivities of the P-S@dioct:DOPC structures at 10^−3^ − 10^−2^ S/cm.

### PEDOT-S and DOPC Mixing Probed with Quenching of Nile Red

**Figure 4:**
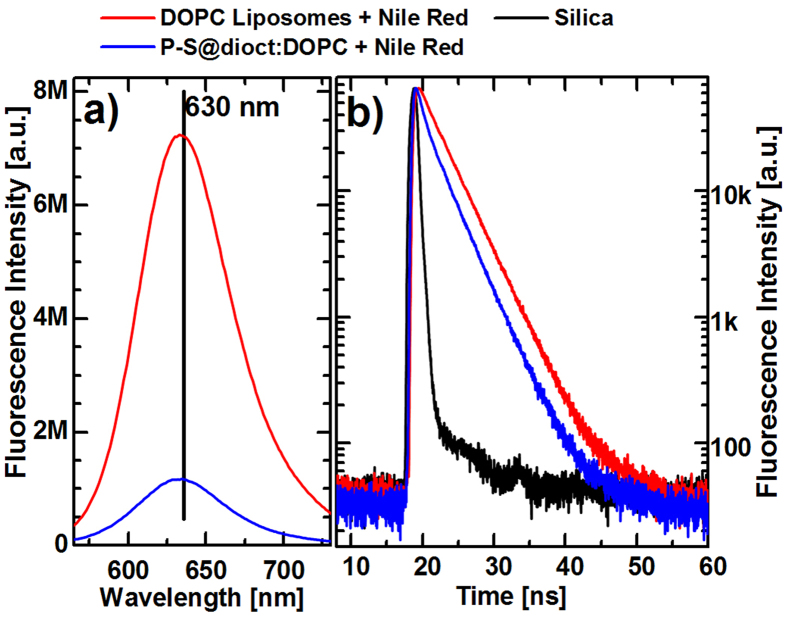
PEDOT-S quenches the fluorescent dye Nile Red in the P-S@dioct:DOPC structures. **a**) When the P-S@dioct:DOPC structures are prepared together with Nile Red, the fluorescence is significantly decreased compared with reference DOPC liposomes stained with Nile Red but without PEDOT-S@dioctyl-ammonium. **b**) The fluorescence decay of Nile Red is quicker (multiexponential) for P-S@dioct:DOPC structures than the monoexponential decay for Nile Red in DOPC liposomes (*τ* = 3.5 ns), which indicates quenching. Excitation wavelength was 550 nm in **a**) and 500 nm in **b**).

The exact nature of the P-S@dioct:DOPC structures cannot be deduced from the data presented above. The hydrophobic PEDOT-S complex may be (i) incorporated into the membranes of the liposomes, (ii) within the interior compartment of the liposome, (iii) or in structures resembling micelles stabilized by phospholipids. A mix of these scenarios is also possible. Therefore we used a fluorescent dye that lights up in hydrophobic environments but that exhibits extremely low fluorescence in water, for spectroscopic evaluation of the mode of integration of PEDOT-S in the liposomes. A slightly modified processing sequence was used in these experiments (see Methods) and the hydrophobic dye Nile Red that has been shown to integrate into the hydrophobic regions of lipid vesicles was used as the fluorescent probe^48^,^49^. In lipid environments, Nile Red emits light with a maximum at about 630 nm and changes of its fluorescence due to the presence of PEDOT-S was analyzed. With an excitation wavelength of 550 nm, we observed a strong reduction of fluorescence in Nile Red—stained liposomes that were prepared together with PEDOT-S@dioctylammonium complexes (Fig. 4a). We also measured the lifetime of the fluorescence using excitation at 500 nm, and noted a change from a monoexponential decay (*τ* = 3.5 ns) to a faster multiexponential decay, in agreement with the reduced quantum yield of fluorescence (Fig. 4b). The fast quenching observed may be caused by excitation quenching of the Nile Red emission by interaction with the metallic PEDOT-S at short distance^50^ or by the Förster resonance energy transfer mechanism. The short distance required between Nile Red and PEDOT-S for both these mechanisms means that PEDOT-S has to be closely coordinated with or within the liposome membrane.

### The Effect of P-S@dioct:DOPC Structures on Shaker K Channels in *Xenopus* Oocyte Membranes

**Figure 5:**
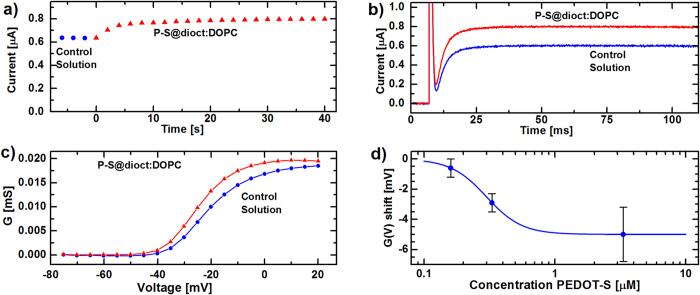
P-S@dioct:DOPC structures affect the voltage dependence of the Shaker K channel. (**a**) 0.33 *μ*M P-S@dioct:DOPC applied to the extracellular solution increases the steady-state K^+^ current at −20 mV. (**b**) Current traces at −20 mV from a holding voltage of −80 mV. Same recording as in (**a**). (**c**) Steady-state K^+^ conductance vs. membrane voltage. (**d**) Dose-response curve of the induced shift. The best-fitted curve is ΔV = ΔVmax / (1 + (K_D_/c)^n^), where ΔVmax = −5.0 mV, K_D_ = 0.30 *μ*M, and n = 3.2. Neither control samples with DOPC liposomes without PEDOT-S@dioctyl-ammonium nor PEDOT-S added to the water phase, resulted in significant shifts. Data shown as mean ± SEM, n = 4 − 10. All concentrations given are based on the PEDOT-S monomer and the pH was 7.4 for all experiments.

Finally, the P-S@dioct:DOPC structures were used in experiments with *Xenopus* oocytes. The question remains if P-S@dioct:DOPC structures can be incorporated into biological lipid membranes. If so, they can potentially affect membrane proteins expressed in cells. Voltage-gated ion channels are well-suited membrane proteins for such experiments; they conduct specific ions across the lipid bilayer, thereby being responsible for nervous impulses and cardiac excitability, and the opening-closing gating is highly sensitive to fixed and mobile charges^51^,^52^,^53^. Charged lipophilic compounds most likely insert into lipid bilayers via its hydrophobic tail; from this position the charged group of the compound electrostatically affects the positively charged voltage sensor S4 of the voltage-sensor domain (VSD) of voltage-gated ion channels, and thereby affects the channel′s open probability and consequently physiological functions^51^,^54^,^55^,^56^,^57^. To investigate the effects of PEDOT-S on ion channels we expressed the Shaker K channel in *Xenopus* oocytes and measured K^+^ currents by the two-electrode voltage-clamp technique. Initial experiments with PEDOT-S in the water phase did not show any effects on the channels, indicating that a delivery system to the membrane is needed. While 0.33 *μ*M P-S@dioct:DOPC (concentrations presented are based on the PEDOT-S monomer) added to the extracellular solution had no effect on uninjected (control) oocytes, it quickly increased the K^+^ current at −20 mV in oocytes expressing Shaker K channels (Fig. 5a,b). The current increase was mainly caused by an alteration of the channel′s voltage dependence (Fig. 5c); 0.33 *μ*M P-S@dioct:DOPC shifted the voltage dependence by −2.9 ± 0.6 mV (n = 10, P = 0.0005). The maximum conductance was not affected. The shift was dose dependent with an apparent maximum shift of −5.0 mV and an apparent K_D_ value of 0.30 *μ*M (Fig. 5d). Application of only DOPC liposomes (at a concentration matching the DOPC concentration for 0.33 *μ*M P-S@dioct:DOPC application) did not shift the voltage dependence of the Shaker K channel (mean shift: −0.3 ± 0.7 mV (n = 7, P > 0.5). Previous studies have suggested that lipophilic charged compounds interact closely (~5 Å) with the VSD of the Shaker K channel^54^. A critical test of this hypothesis is that introduction of two positively charged arginines at the extracellular end of S4 (356R and 359R) affects the effect of lipophilic charged compounds^56^. This mutation completely abolished the shift effect of 0.33 *μ*M P-S@dioct:DOPC (mean shift: +0.1 ± 0.2 mV, n = 6, P > 0.5), suggesting that the complex with PEDOT-S is located in or close to the lipid membrane close to the channel′s voltage sensor. However, the effect of the mutation on P-S@dioct:DOPC sensitivity was opposite to that found for polyunsaturated fatty acids^54^,^56^; introduction of two positively charged residues at the extracellular end of the voltage sensor S4 increased the effect of polyunsaturated fatty acids while it decreased the effect of PEDOT-S. If we assume electrostatic effects and that the active form of the compounds is negatively charged, this suggests different orientations relative the voltage sensor S4 for PEDOT-S and the polyunsaturated fatty acid.

## Discussion

We have hydrophobized PEDOT-S by creating electrostatic complexes of PEDOT-S and alkyl-ammonium salts, which can be dissolved in organic solvents with preserved doping and electronic conductivity. With hexadecyl-trimethyl-ammonium, a complex with slightly higher conductivity was obtained compared to with dioctyl-ammonium. The exact origin of this difference is currently unknown. However, the complexes that we created enabled us to form conductive hybrid structures with PEDOT-S@alkyl-ammonium inserted into DOPC phospholipid liposomes, possibly within the bilayer membrane. We demonstrated effective quenching of the membrane dye Nile Red when it was included in the P-S@dioct:DOPC structures, which shows that PEDOT-S is closely coordinated in or on the liposome membranes. The QCM-D experiments revealed that these structures adsorb onto supported lipid bilayers, and we were able to measure currents through them with two distinct techniques. This is a first demonstration of conductive lipid membranes with potential to carry electronic currents across the membrane as well as laterally over longer distances. Soft electrical interfaces like conductive lipid bilayers may have applications within many fields, such as biosensing, controlled release, directed cell growth and probing of bioprocesses - just to mention a few. We also demonstrate in this work that the produced structures can function as vehicles to deliver the metallic polythiophene PEDOT-S to the membranes of living cells, possibly by membrane fusion. When this was utilized on *Xenopus* oocytes with Shaker K channels expressed, the gating of these channels was modified, which serves as an example that it is possible to access and control structures in the membranes of living cells with the electronic lipid:hybrid structures that we created. This is a major step towards incorporating these self-doped conductive polyelectrolytes into lipid membranes and thus create a new pathway to make conductive MMS and to achieve electronic access to redox active elements in biological systems.

## Methods

### Chemicals

Nonyl-amine, dioctyl-amine, tetraethyl-ammonium chloride, tetrabutyl-ammonium fluoride and hexadecyl-trimethyl-ammonium chloride were used to prepare 2.5 mM alkyl-ammonium salt solutions in MilliQ water that were adjusted to pH 4–9, depending on the experiment. The amines, ammonium salts and the Nile Red membrane dye were purchased from Sigma Aldrich. Unsaturated DOPC dissolved in chloroform was purchased from Avanti Polar Lipids. The PEDOT-S was synthesized at the organic chemistry department at Linköping University, in accordance with previously described protocols^27^,^31^,^43^.

### Preparation of PEDOT-S@alkyl-ammonium complexes and P-S@dioct:DOPC structures

200 *μ*L of PEDOT-S in MilliQ (1 mg/mL) was added to 1.3 mL of the prepared alkyl-ammonium salt solution (2.5 mM), and the mixture was heated briefly to ~60 °C resulting in a blue precipitate that was centrifuged (10,000 g, 5 min) whereafter the supernatant was removed. To rinse away excess alkyl-ammonium salt, the pellet was resuspended in MilliQ pH-adjusted for the experiment, and the centrifugation step was repeated. Thereafter, was the pellet dried with N_2_ gas and dissolved in 200 *μ*L chloroform:methanol (2:1), thus giving a final PEDOT-S concentration of 1 mg/mL PEDOT-S (precipitates from nonyl- and tetrabutyl-ammonium had limited solubilities and tetraethyl-ammonium did not dissolve at all). PEDOT-S@dioctyl-ammonium complexes were used to make P-S@dioct:DOPC structures. 200 *μ*L solution with this complex was mixed with 2 mL DOPC (1 mg/mL) in chloroform:methanol (2:1). A lipid cake was formed by blowdrying with N_2_ gas until the solvent was completely evaporated. The lipid cake was suspended in 2 mL PBS at pH 7.4 (this pH was used for all subsequent experiments with the structures). The suspension was stirred (1100 rpm) for 30 min, stored 60 min in 4 °C and stirred (1100 rpm) for 30 min, before it was extruded with polycarbonate membranes having 100 nm orifices. Reference liposomes were prepared in the same way, but without adding the PEDOT-S@dioctyl-ammonium complex.

### UV-Vis spectroscopy and electrical characterization of PEDOT-S@alkyl-ammoinum complexes

The PEDOT-S@alkyl-ammonium complexes were diluted to 100 *μ*g/mL in chloroform:methanol (2:1) and the absorbance was measured with a UV-Vis spectrophotometer in the range 400–800 nm for the various samples. Films were blade coated on microscope glass slides from concentrated PEDOT-S@dioctyl-ammonium and PEDOT-S@hexadecyl-ammonium solutions, respectively, and the film thicknesses were measured with a profilometer (~100 nm). Electrodes seperated by 1 cm were prepared on the films with silver paste and currents were measured with two probe measurements during 0–1 V voltage sweeps with a Keithley 4200 parameter analyzer. The conductivities were then calculated from the film geometries and the slopes of the obtained I-V curves.

### QCM-D

QCM-D was performed with SiO_2_ sensors on a Q-Sense E4 instrument and an Ismatec Reglo Digital M2-2/12 pump, set to 100 *μ*L/min. The presented graphs are the 5th overtones. In the first experiment, a supported lipid bilayer was first formed by introducing DOPC reference liposomes (500 *μ*g/mL). Thereafter, the sample solution with P-S@dioct:DOPC structures (500 *μ*g/mL DOPC, 50 *μ*g/mL PEDOT-S) was introduced during 18 min, after which the system was rinsed with PBS buffer for 40 min. In the second experiment, no lipid bilayer was created in beforehand, but the sample solution was introduced directly to the sensor. After running the sample for 16 min, the system was rinsed with PBS buffer for 50 min.

### AFM

A supported lipid bilayer was formed on a SiO_2_ substrate with DOPC liposomes (500 *μ*g/mL) during 10 min. The surface was then rinsed by submerging it in PBS buffer for 10 min and the sample solution with P-S@dioct:DOPC structures (500 *μ*g/mL DOPC, 50 *μ*g/mL PEDOT-S) was then added on top. After 15 min incubation, the surface was rinsed by submerging it in MilliQ for 30 s. After drying in ambient room conditions, the surface was imaged in a Dimension 3100 SPM system.

### Electrical characterization with nanoelectrodes

Au nanoelectrodes on silicon surfaces with 10 fingers from each side á 20 *μ*m and electrode gaps of 100 nm were produced at Chalmers University of Technology in Gothenburg. In the same way as in the AFM experiment described above, supported lipid bilayers were created on these, on which P-S@dioct:DOPC structures then were adsorbed. Two types of control samples were prepared: (i) pure supported lipid bilayers on the nanoelectrodes and (ii) supported lipid bilayers on the nanoelectrodes, treated with PEDOT-S dissolved in MilliQ (100 *μ*g/mL) during 15 min incubation time followed by 30 s rinsing with MilliQ. The conductivities for the various samples were measured with two probe measurements using a Keithley 4200 parameter analyzer.

### C-AFM

Pt films were sputtered on SiO_2_ substrates. On these were P-S@dioct:DOPC structures added (500 *μ*g/mL DOPC, 50 *μ*g/mL PEDOT-S), which had been prepared with polycarbonate membranes having 400 nm orifices in the extrusion process. After 15 min incubation, the surfaces were rinsed by submersion in MilliQ for 30 s. Local electrical characterization was then conducted in a Dimension 3100 (Bruker) microscope with a Nanoscope IV controller equipped with a C-AFM module (1 nA/V current sensitivity). Commercial Pt/Ir coated silicon probes having nominal spring constant of 0.2 N/m were used to perform imaging and measure local current-voltage characteristics in contact mode, by applying load forces of 2–5 nN.

### Quenching

For the quenching experiments, pure chloroform was used as solvent and 10 *μ*L Nile Red (1 mg/mL in chloroform) was added in the preparation of the P-S@dioct:DOPC lipid cake. Also, the samples were ultra-sonicated for 5 min, before extrusion. The fluorescence measurements were acquired using a Horiba Jobin Yvon Fluoromax 4 spectrofluorometer. Lifetime data were collected with a time-correlated single photon counting spectrometer (Mini-*τ*, Edinburgh Instruments). A longpass filter was used in order to absorb the scattered light above 540 nm and a gray filter having absorbance 1.0 was used in order to attenuate the laser intensity. All the measurements have been performed in air.

### Expression of ion channels and electrophysiological recordings

Experiments were performed on the Shaker H4 channel^58^ and the Shaker 3R channel (i.e. 356R/359R;^56^) made incapable of fast inactivation by the Δ(6–46) deletion^59^. Mutagenesis, cRNA synthesis, *Xenopus* oocyte preparation, cRNA injection and oocyte storage follows the procedures described previously^54^,^55^. Animal experiments were approved by the local Animal Care and Use Committee at Linköping University. Ion currents were recorded by the two-electrode voltage-clamp technique (CA—1B amplifier, Dagan Corporation, Minneapolis, MN), Digidata^TM^1440A digitizer and pClamp^TM^10 software (Molecular Devices, Union City, USA) 1–6 days after injection of RNA. The amplifier′s leak and capacitance compensation were used and currents low-pass filtered at 5 kHz. All experiments were done at room temperature (20–23 °C) and at pH 7.4. The holding voltage was set to −80 mV and steady-state currents measured at voltages between −80 and +50 mV. The control solution contained (in mM): 88 NaCl, 1 KCl, 15 HEPES, 0.4 CaCl_2_, and 0.8 MgCl_2_. pH was adjusted to 7.4 with NaOH yielding a final sodium concentration of ~100 mM. Pure control solution was added using a gravity-driven perfusion system. All chemicals were from Sigma-Aldrich (Stockholm, Sweden). The K conductance G_K_(V) was calculated as G_K_(V) = I_K_ / (V–V_rev_), where I_K_ is the steady-state current at the end of a ~80-ms pulse, V the absolute membrane voltage, and V_rev_ the reversal potential for the K channel, set to −80 mV. The PEDOT-S-induced shift of the G_K_(V) curve was quantified at the 10% level as previously described^57^.

### Statistical analysis

Average values are expressed as mean ± SEM. Statistical analyses in mean values for G(V) shifts were carried out using two-tailed one sample t-test where the mean values were compared to a hypothetical value of 0. P < 0.05 is considered as significant.

## Additional Information

**How to cite this article**: Johansson, P. K. *et al.* Electronic polymers in lipid membranes. *Sci. Rep.* **5**, 11242; doi: 10.1038/srep11242 (2015).
